# The "Colony Treatment" of Epileptics

**Published:** 1900-09-29

**Authors:** 


### THE "COLONY TREATMENT" OF EPILEPTICS.

A good deal of experience has now been accumulated in regard to the effect of treating epileptics in what are called "farm colonies," that is, of dealing with them away from their homes, and apart from patients suffering from other forms of disease, and of treating them principally by *régime* and properly apportioned work and occupation. Dr. Aldren Turner recently gave an account of the results which had been met with among the patients so treated at the farm colony opened in the year 1893 by the National Society for the Employment of Epileptics at Chalfont St. Peter, where a register is kept of all fits, of the work allotted to each colonist, and of the influence which one or other kind of work may appear to have upon him. In considering these results, however, it must be remembered that care is taken in the selection of cases for admission to the colony. It has been found advisable, for example, to refuse all those with obvious evidence of organic cerebral disease; those in whom epilepsy developed late in life; those who have been for prolonged periods in the workhouse infirmary, and those who are manifestly insane. The cases preferred are those of young adults in whom the disease is of comparatively recent origin. Bearing this in mind, it is to be noticed that increased experience favours the view that the early treatment of epilepsy ought to be institutional in its methods, so that regular and congenial employment may be found in garden, fields, orchards, or workshop, under supervision; that the mode of life may be well ordered and regular, with avoidance of excitement and abstinence from alcoholic liquors; that the nourishment may be abundant but of a simple character; and that the medicinal treatment may be reduced to a minimal amount, namely, half a drachm of bromide of potassium at bedtime. As to the effect of the treatment upon the fits, it seems that in most cases an average frequency of seizures is after a time arrived at, which is maintained with surprising regularity. Clearly the fits are not cured, although it would appear that in the majority they are diminished both in frequency and severity. The mental state, however, has in the great majority been maintained, the usual downward tendency towards dementia having been manifested in only a very small minority, while as to physical condition there has been distinct improvement. Much stress is laid upon the advantage which is gained by placing young epileptics on such farm colonies, where they can work under supervision, the advantage of the institutional colony over the ordinary farm lying especially in the regularity of the life and the supervision of the work. Certainly the experience gained at the Chalfont Colony shows that it provides the most satisfactory means of dealing with epileptics in the humbler walks of life, and it seems reasonable to suggest that at such colonies arrangements should be made for the reception of private patients. It has to be recognised that the disease is not often cured. But the utility of the treatment is very marked in other ways. It is a great thing to provide employment for a class of the community who find great difficulty in finding work on account of their malady, and to show that, "if interest is taken in his work, in his mode of life, and in his amusements, the confirmed epileptic is capable of becoming quite a useful citizen."

